# Supplementary material for: Automatically Defining Protein Words for Diverse Functional Predictions Based on Attention Analysis of a Protein Language Model
Source: Adv Sci (Weinh). 2026 Feb 5;13(21):e21970. doi: 10.1002/advs.202521970 (PMC13073253; doi:10.1002/advs.202521970)
Supplement: Supplementary file 1 — Supporting File: advs74259‐sup‐0001‐SuppMat.pdf. [file ADVS-13-e21970-s001.pdf]

Supporting Information for
Automatically Defining Protein Words for Diverse Functional Predictions Based on Attention Analysis of a Protein Language Model

Hedi Chen[a,1], Jingrui Zhong [a,1], Xiaochun Zhang [a,1], Jingke Chen [a,1], Lin Guo [a,1], Xiaoliang Xiong [a,1], Xiaonan Zhang[b], Xiangyu Liu [a], Bailong Xiao [a], Boxue Tian[a*]

Boxue Tian
Email: boxuetian@mail.tsinghua.edu.cn

This PDF file includes:

Supporting text
Figures S1 to S13
Tables S1 to S11

Figures

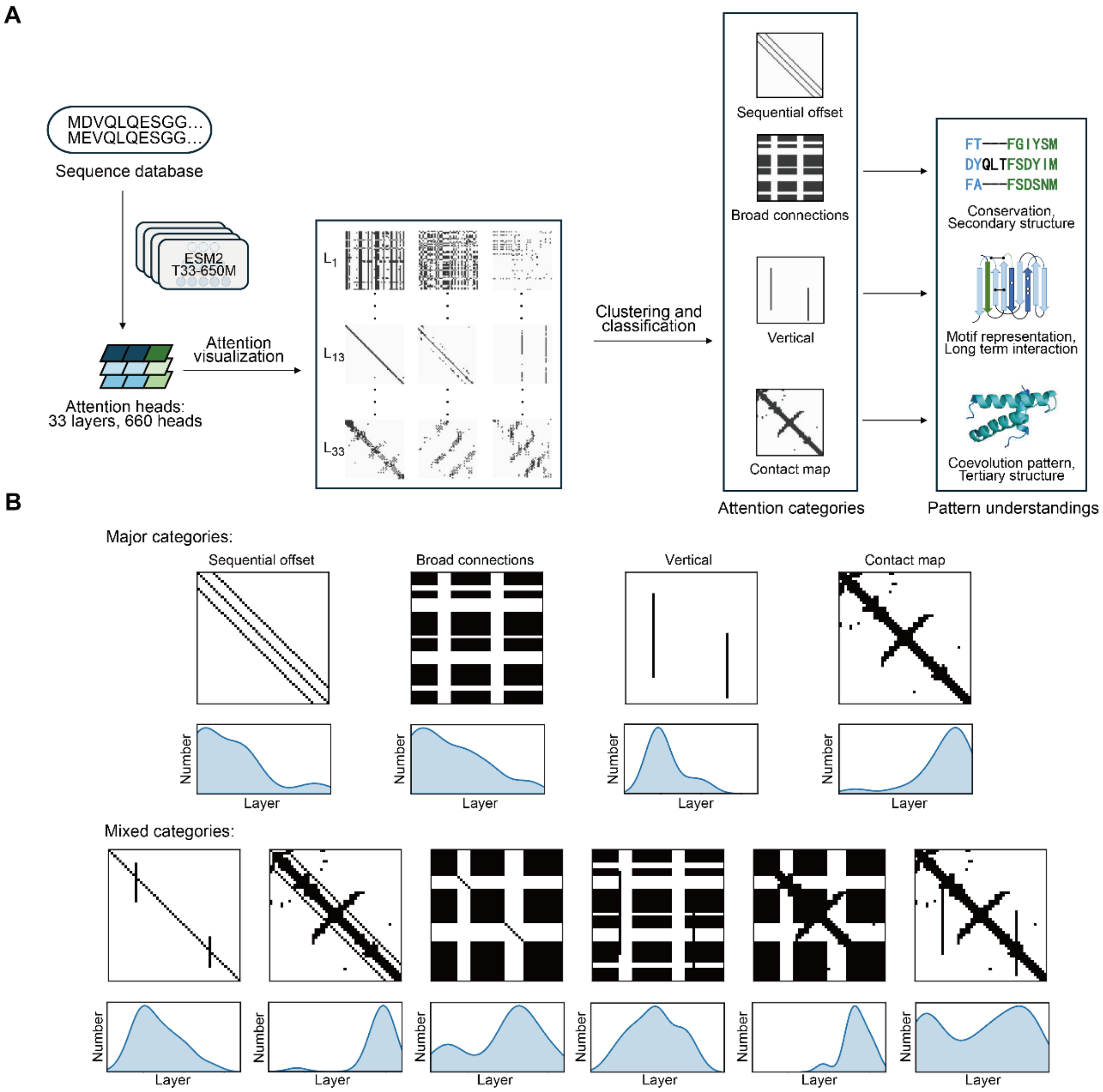

Fig. S1. Attention categories of the PLM (ESM2). (**A**), Four major attention head categories. In the first step, the protein sequence is passed to ESM2 T33-650M model for attention prediction. Based on the observations of from >1,000 sequences, we identified 4 major attention categories: sequential offset, broad connections, vertical (key residues) and contact map. The categories of attention heads were consistent across different input sequences. (**B**), An overview of all attention head categories. The top images depict the categories and the bottom images represent the distribution of the attention heads for the same category across different layers. The contact map categories are mostly located in Layers 31-33; the broad connection categories are mostly located in Layers 1-10.

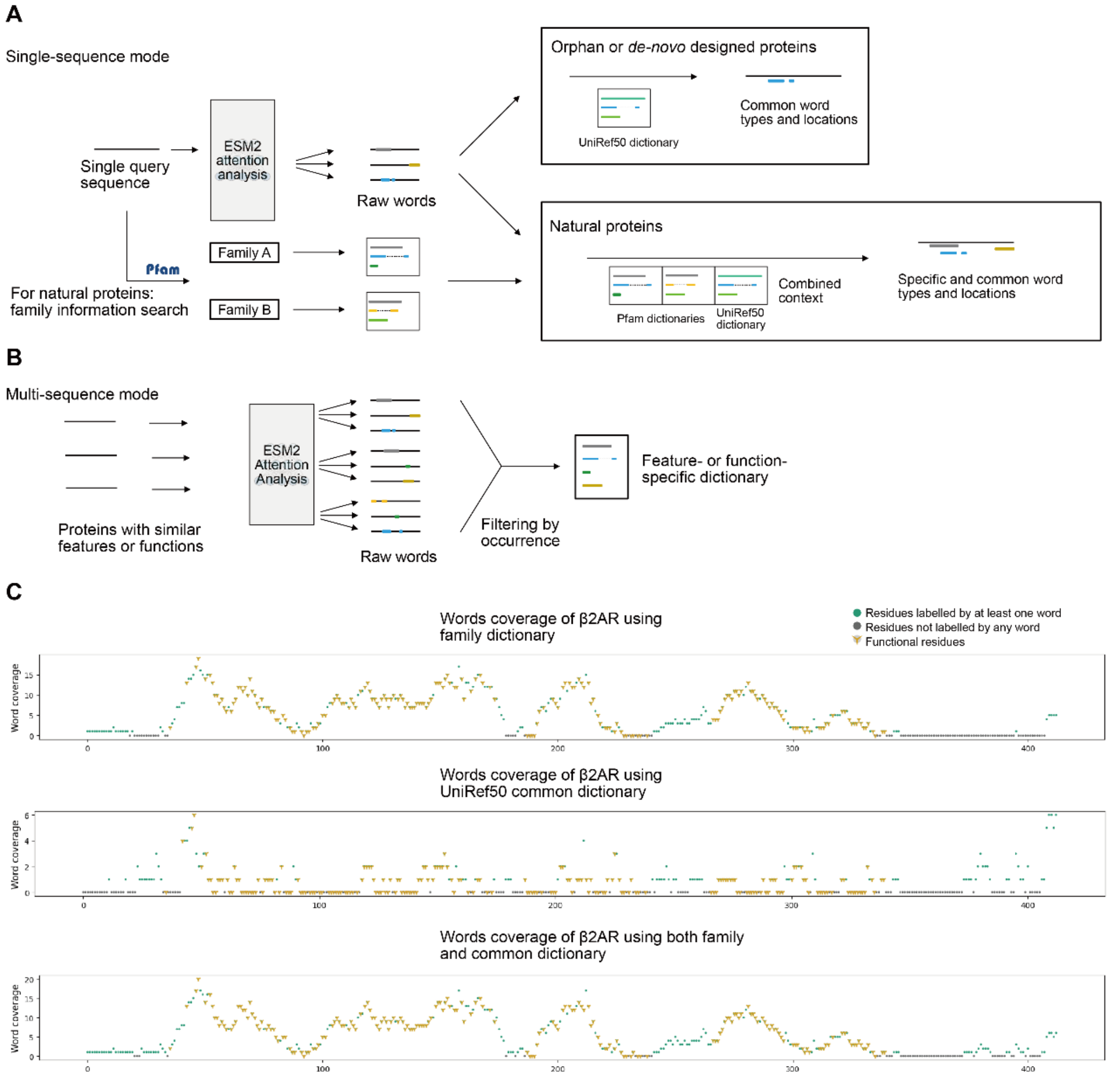

Fig. S2. Single- and multi-sequence modes of Protein Wordwise. (**A**), Flowchart for the single-sequence mode of Protein Wordwise. For an analyte protein sequence lacking Pfam information, protein words were predicted by matching raw words predicted by the Louvain algorithm and the UniRef50 dictionary. For analyte sequences having known UniProt ID and Pfam, the UniRef50 and all suitable Pfam dictionaries are used for matching. (**B**), Flowchart for the multi-sequence mode of Protein Wordwise. Protein words are predicted from all sequences using the Louvain algorithm, stratified by length; only protein words with the highest occurrences (for each length bin) are selected for dictionary construct ion (Methods). (**C**), An example of functional residue coverage and functional residue prediction using solely a Pfam dictionary (Pfam PF00001), using solely a UniRef50 dictionary, or using both dictionaries.

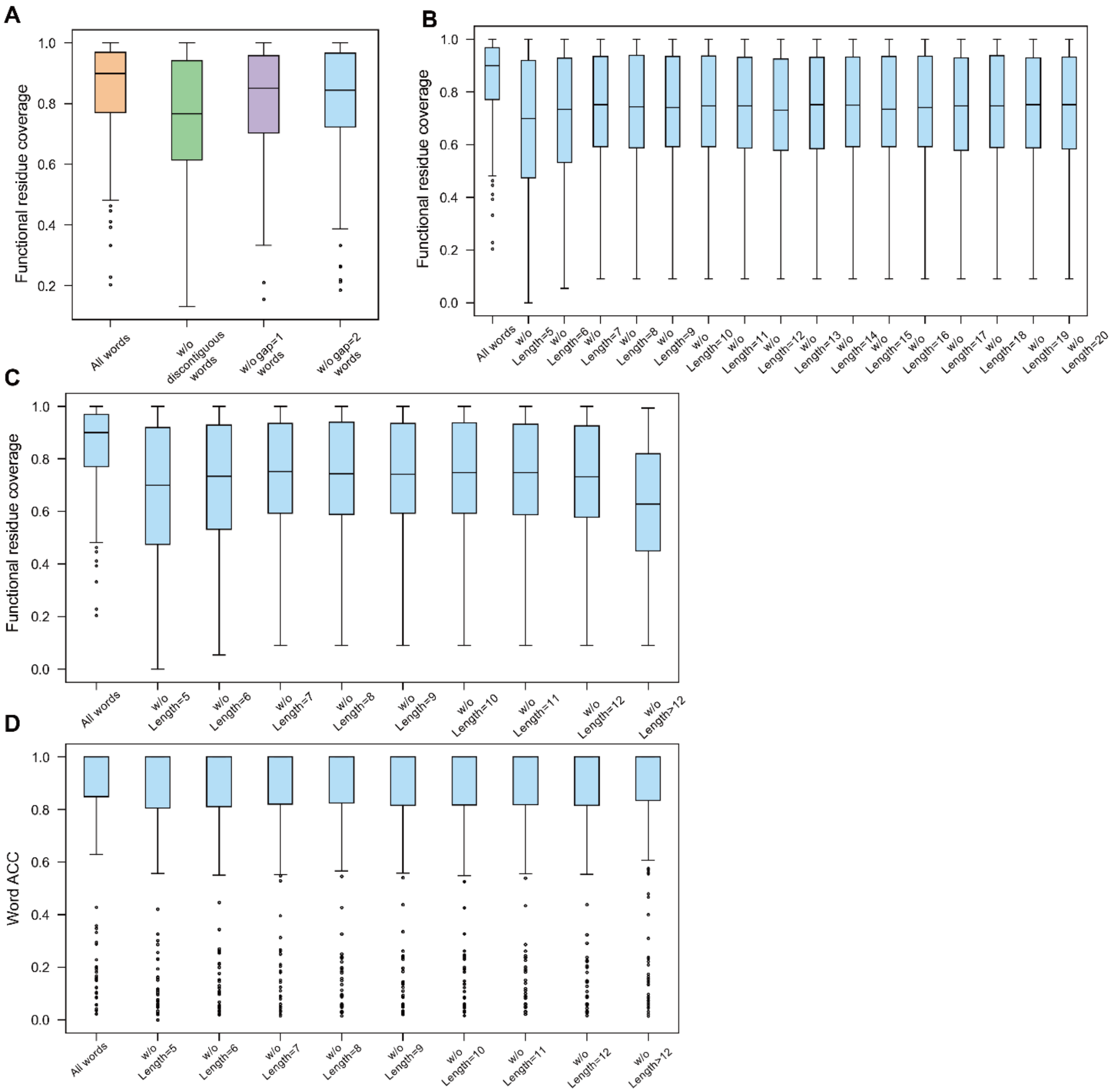

Fig. S3. Ablation studies of Protein Wordwise using the DMS dataset. (**A**), Functional residue coverage performance of Protein Wordwise after removing discontiguous words, discontiguous words with one gap, or discontiguous words with two gaps. (**B**), Functional residue coverage performance of Protein Wordwise after removing words of differing lengths as indicated. (**C**), After merging words of length greater than 12, the functional residue coverage performance of Protein Wordwise upon removing words with different lengths. (**D**), The accuracy of Protein Wordwise upon removing words with different lengths in the DMS dataset.

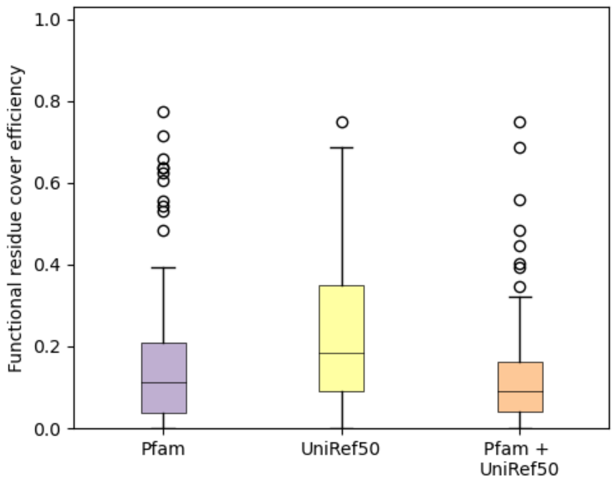

Fig. S4. Efficiency comparison for Pfam and UniRef50 dictionaries, as well as the combined version. Higher efficiency score indicates that the predicted functional word table size per functional residue it covered is smaller, and thus the functional words prediction has less redundancy. In the DMS dataset, the Protein Wordwise using both dictionaries reached a mean cover efficiency of 0.120. Using Pfam and UniRef50 dictionaries solely results in efficiency scores of 0.154 and 0.232. The cause of lower efficiency in the combined version is the overlap of correctly covered functional residues between words from the two sources.

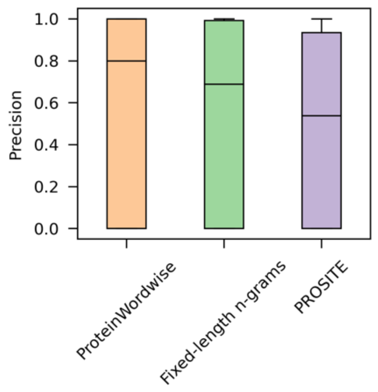

Fig. S5. Residue-level precision comparison among different annotation strategies. Boxplots show the distribution of residue-level precision for ProteinWordwise, a fixed-length n-gram baseline without word segmentation, and the motif-based method PROSITE. Median precision values are 0.800 for ProteinWordwise, 0.688 for fixed-length n-grams, and 0.538 for PROSITE, indicating that ProteinWordwise achieves superior precision in functional residue identification. This result highlights the advantage of protein word representations over both motif annotations and non-segmented sequence baselines.

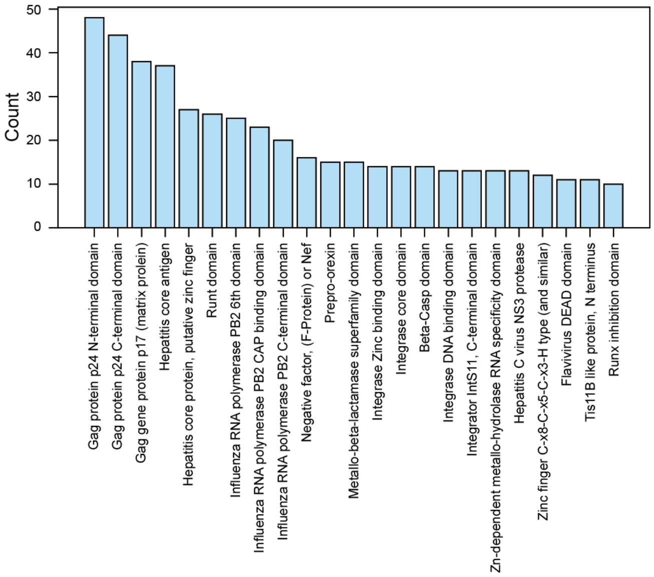

Fig. S6. Distribution of Pfam domains among the 1,202 proteins containing MHC peptides and having experimental 3D structures. The top three Pfam domains were all Gag, with 48, 42, and 38 proteins, respectively. Gag is a structural protein found in HIV-1 and other retroviruses.

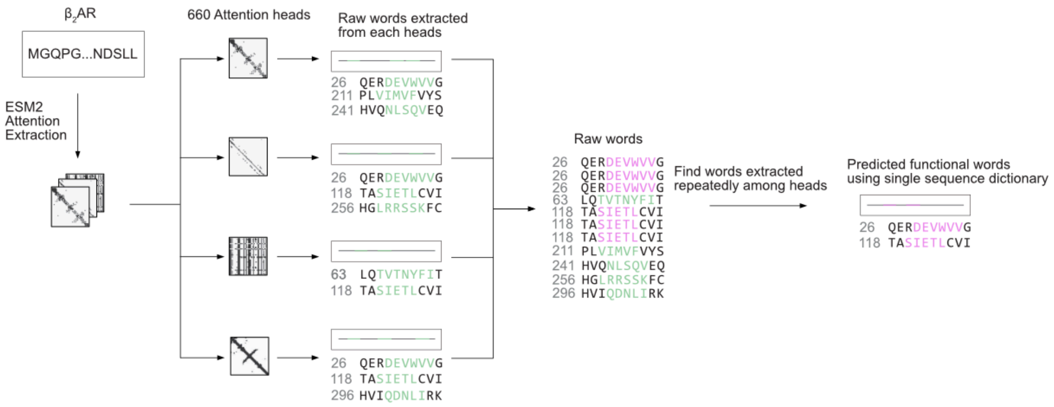

Fig. S7. A schematic diagram of single sequence dictionary. Initially, we constructed a "single sequence dictionary" from raw words within a single sequence that appeared repeatedly across different attention heads. We then used the words in this single sequence dictionary as predicted functional words. However, using this single sequence dictionary led to the elimination of many potentially useful words. To address this, we decided to use the Pfam dictionary and UniRef50 dictionary to "rescue" the words that the single sequence dictionary had removed.

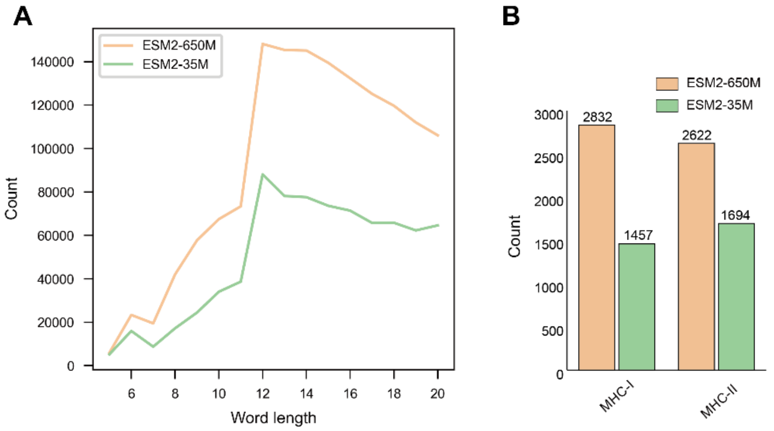

Fig. S8. Performance comparison of the UniRef50 dictionary for ESM2_35M and ESM2_650M. (**A**), The number of words at different word lengths are similar for ESM2_35M and ESM2_650M. (**B**), ESM2_650M achieved the higher overall MHC peptide count (MHC1=2,832, MHC2=2,622) than ESM2_35M (MHC1=1,457, MHC2=1,694). ESM2_35M matched MHC peptides with a slightly lower number of unique words (251.2 words per MHC peptide) compared to ESM2_650M (268.2 words per MHC peptide).

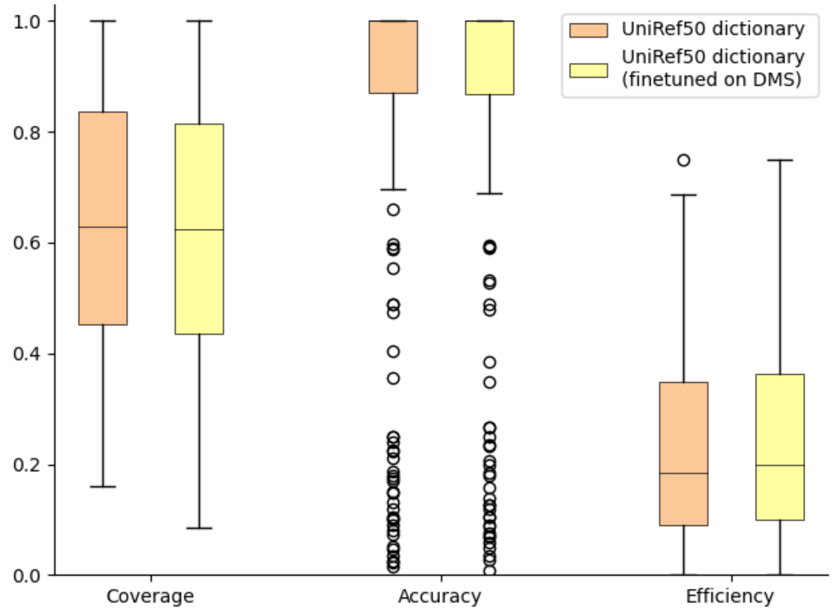

Fig. S9. Performance comparison of the UniRef50 dictionary for parameter sets generated from Beta2AR and DMS. The magnitude of change is small for all metrics (coverage, accuracy and efficiency), indicating that optimization on the DMS dataset did not obviously improve overall performance.

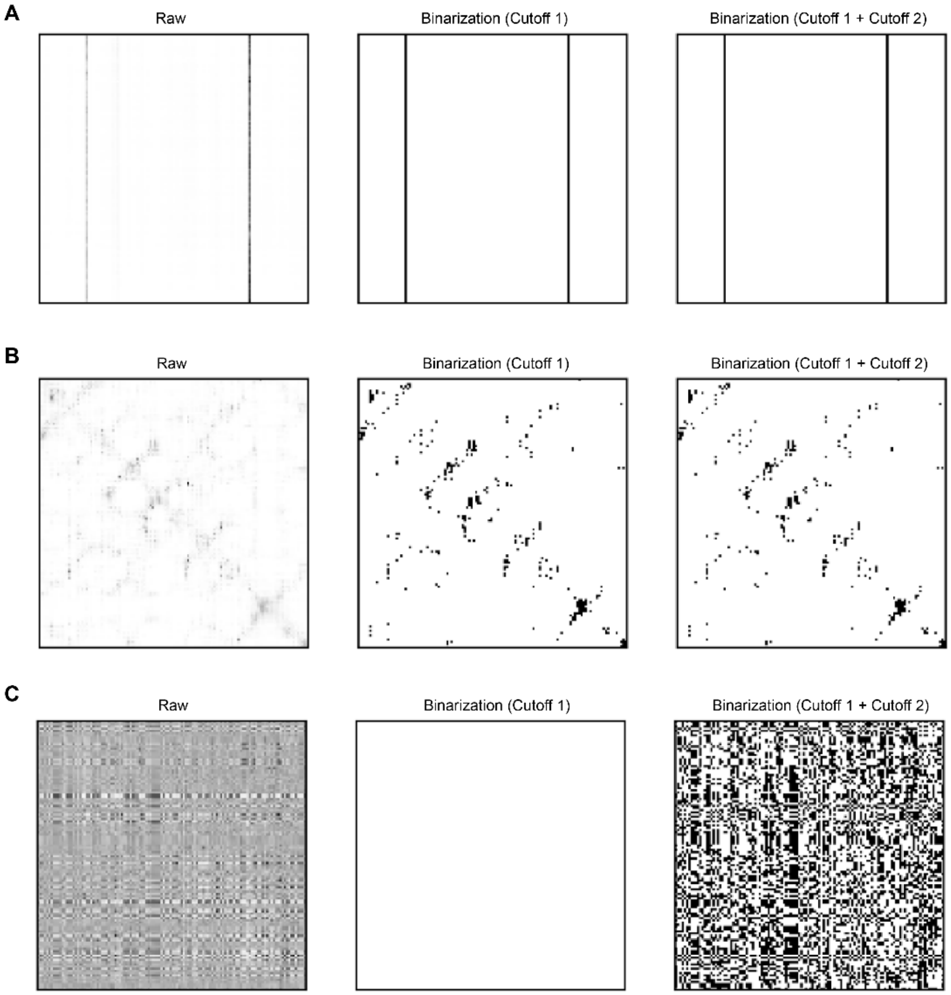

Fig. S10. Illustration of the use of cutoff values for our binarization strategy. (**A**)- (**B**), Using Cutoff_1, we achieved reasonable binarization performance in attention heads such as vertical lines and contact matrices. (**C**), In broadly connected heads, Cutoff_1 discarded a substantial portion of signal. To address this, we introduced Cutoff_2, which removed low-value data proportionally and preserved information in these attention heads.

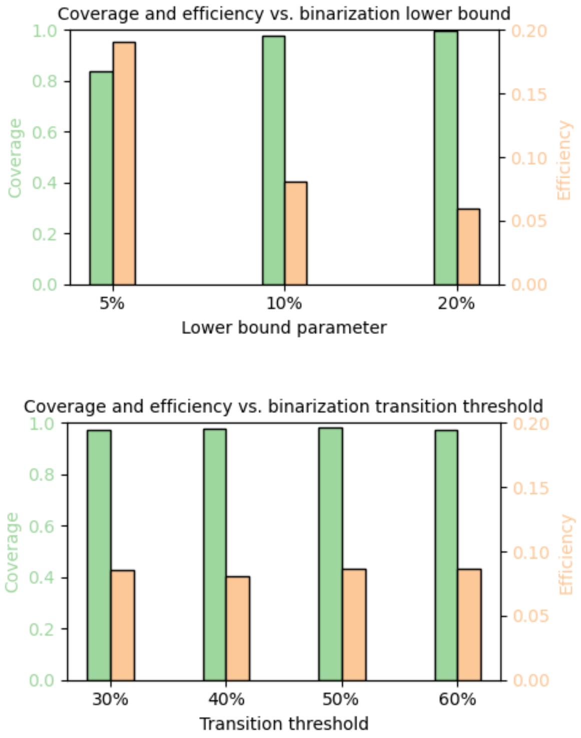

Fig. S11. Coverage and efficiency under different word binarization parameter settings. Bar plots compare coverage and efficiency scores of Protein Wordwise under varying binarization parameters, including lower bound and transition threshold to use the upper parameter method. The baseline configuration, lower bound parameter 10% and upper bound transition threshold 40%, achieves a coverage of 0.979 and an efficiency of 0.081.

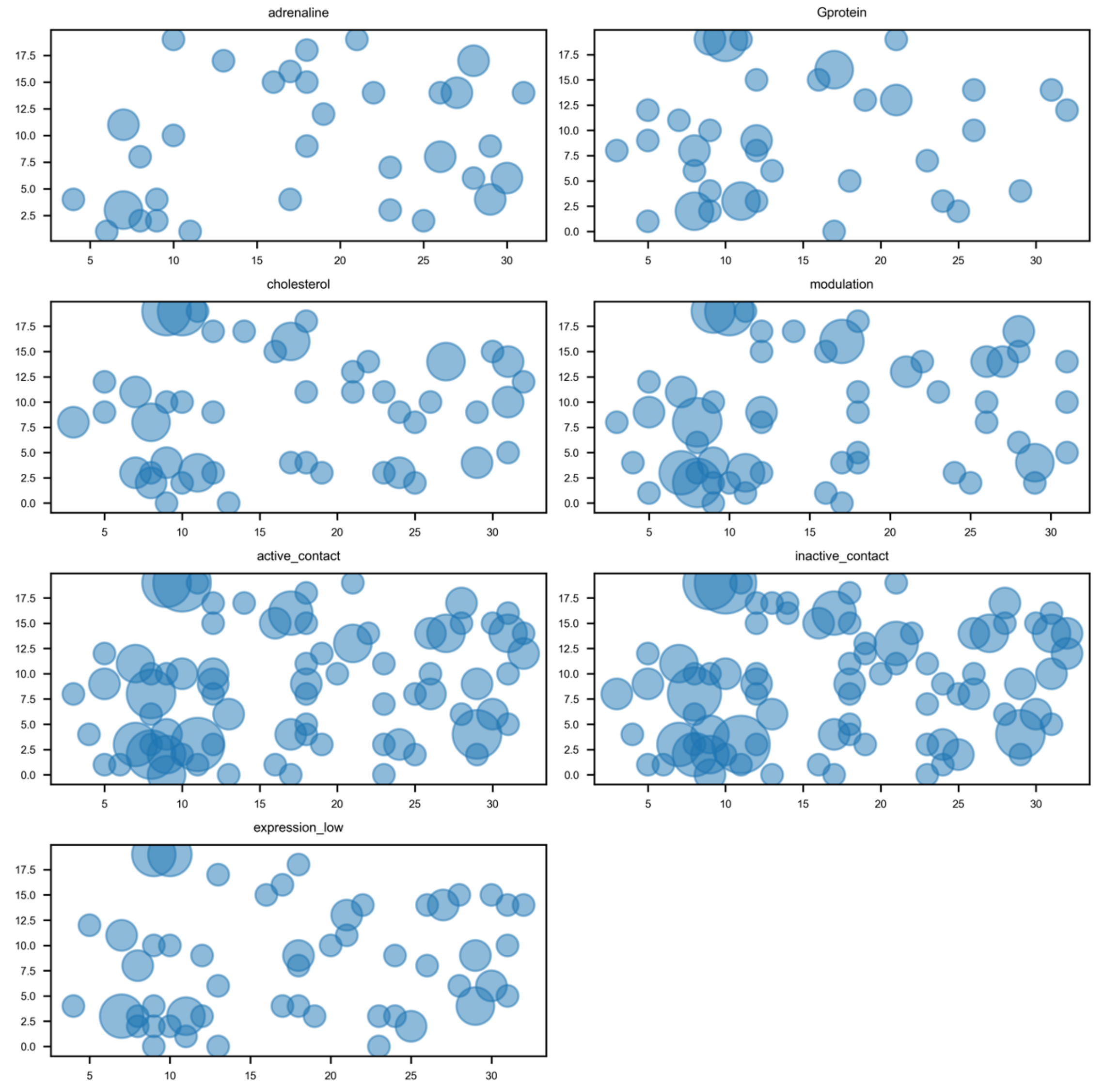

Fig. S12. Layer- and head-wise distribution of functional word signals in the human β2-adrenergic receptor ($β_2$AR). Scatter plots show the contributions of individual attention heads across the 33 layers of ESM2 to different experimentally validated functional words in $β_2$AR, including the adrenaline-binding pocket, G-protein coupling interface, cholesterol-binding motifs, modulation/allosteric sites, active-state contacts, inactive-state contacts, and low-expression–associated regions. Each point represents a single attention head, with the x-axis indicating the layer index and the y-axis denoting the relative contribution strength. Point size encodes the number of residues assigned to the corresponding functional word by that head.

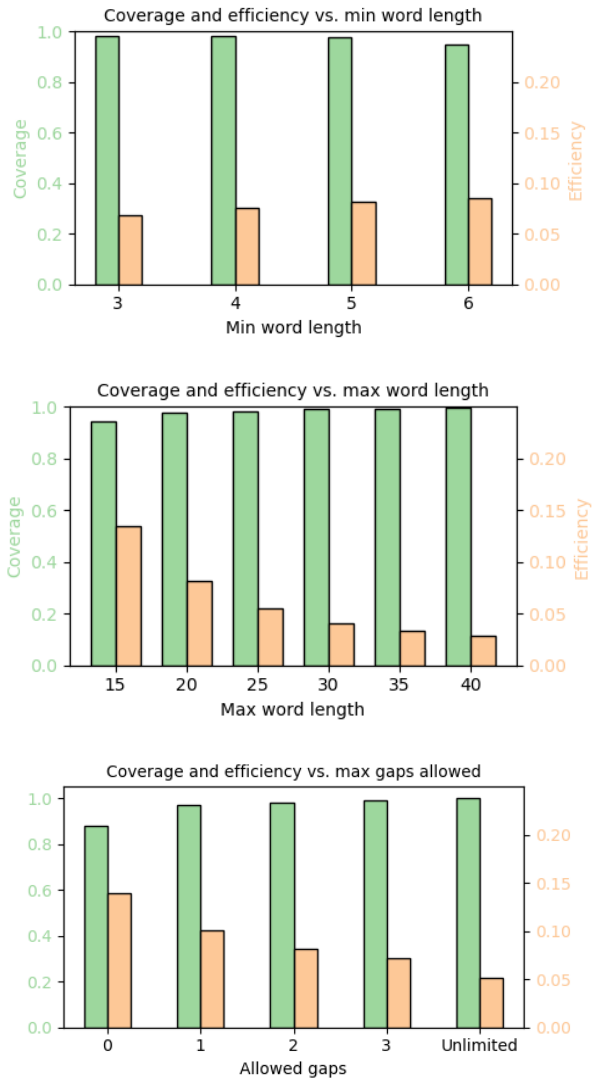

Fig. S13. Coverage and efficiency across different word-length ranges and gap constraints. Bar plots compare coverage and efficiency scores of ProteinWordwise under varying lower and upper bounds of word length and maximum gap number. The baseline configuration achieves a coverage of 0.979 and an efficiency of 0.081. Deviations from this parameterization either reduce coverage by more than 0.03 or substantially impair efficiency, while yielding only marginal coverage gains of approximately ≤ 0.01. These results demonstrate that the selected parameters effectively minimize redundancy while preserving near-complete functional coverage.

Table S1. Tunning hyperparameters based on the β_2AR experimental data.

Contiguous short word percentage*	Contiguous long word percentage*	Discontiguous short word percentage*	Discontiguous long word percentage*	Number of words	Functional residue coverage	Word accuracy
0.025	0.0025	0.000	0.0000	108	0.908	0.907
0.025	0.0050	0.000	0.0000	128	0.923	0.852
0.025	0.0075	0.000	0.0000	161	0.954	0.745
0.050	0.0025	0.000	0.0000	113	0.908	0.903
0.050	0.0050	0.000	0.0000	133	0.923	0.850
0.050	0.0075	0.000	0.0000	166	0.954	0.747
0.050	0.0100	0.000	0.0000	190	0.969	0.726
0.050	0.0150	0.000	0.0000	230	0.990	0.683
0.050	0.0200	0.000	0.0000	250	1.000	0.660
0.050	0.0250	0.000	0.0000	262	1.000	0.653
0.050	0.0300	0.000	0.0000	269	1.000	0.651
0.075	0.0025	0.000	0.0000	119	0.908	0.874
0.075	0.0050	0.000	0.0000	139	0.923	0.827
0.075	0.0075	0.000	0.0000	172	0.954	0.733
0.150	0.0100	0.000	0.0000	204	0.969	0.711
0.150	0.0150	0.000	0.0000	244	0.990	0.672
0.150	0.0200	0.000	0.0000	264	1.000	0.652
0.150	0.0250	0.000	0.0000	276	1.000	0.645
0.150	0.0300	0.000	0.0000	283	1.000	0.643
0.100	0.0100	0.000	0.0000	200	0.969	0.715
0.100	0.0150	0.000	0.0000	240	0.990	0.675
0.100	0.0200	0.000	0.0000	260	1.000	0.654
0.100	0.0250	0.000	0.0000	272	1.000	0.647
0.100	0.0300	0.000	0.0000	279	1.000	0.645
0.025	0.0025	0.015	0.0010	167	0.959	0.928
0.025	0.0025	0.015	0.0020	186	0.979	0.930

0.025	0.0025	0.010	0.0025	191	0.985	0.927
0.025	0.0025	0.025	0.0010	174	0.969	0.931
0.025	0.0025	0.025	0.0015	183	0.985	0.929
0.025	0.0025	0.025	0.0020	193	0.985	0.933
0.025	0.0025	0.025	0.0025	201	0.990	0.930
0.025	0.0025	0.025	0.0050	232	0.990	0.922
0.025	0.0025	0.025	0.0075	259	0.990	0.896
0.025	0.0025	0.025	0.0100	273	0.990	0.894
0.025	0.0025	0.020	0.0015	177	0.979	0.927
0.025	0.0025	0.020	0.0020	187	0.979	0.930
0.025	0.0025	0.020	0.0025	195	0.985	0.928
0.025	0.0025	0.100	0.0025	228	0.995	0.899
0.025	0.0025	0.100	0.0050	259	0.995	0.896
0.025	0.0025	0.100	0.0075	286	0.995	0.874

* Length for short words ranges from 5 to 6; length for long words ranges from 7 to 20. The UniRef50 dictionary was not included in these predictions.

Table S2. The 12-degenerated residue types defined in this study

Residue type	One code amino acid	Notes
a	D,E	Negatively charged
b	K,R	Positively charged
C	C	Cysteine
h	L,I,V	Hydrophobic (branched chain)
H	H	Histidine
M	M	Methionine
n	N,Q	Amino acids with an amide group
o	S,T	Amino acids with hydroxyl
s	A,G	Hydrophobic (short chain)
P	P	Proline
Y	Y	Tyrosine
@	F,W	Aromatic

Table S3. Functional residue coverage and word accuracy comparison among different schemes for residue degeneration for β_2AR

Degenerate residue scheme	Functional residue coverage	Word accuracy
4	0.918	0.752
**12**	**0.985**	**0.929**
20	0.918	0.862

The UniRef50 dictionary was not included in these predictions.

Table S4. Tunning of schemes for residue degeneration based on the β_2AR experimental data

Residue	4-type codes	Remarks	12-type codes	Remarks
D	a	Negatively charged	a	Negatively charged
E				
K	b	Positively charged	b	Positively charged
R				
H			H	Histidine
A	h	Hydrophobic	s	Hydrophobic, with small sidechain
G				
I			h	Hydrophobic
L				
V				
M			M	Methionine
P			P	Proline
F			@	Aromatic
W				
S	p	Polar	o	Polar, with hydroxyl
T				
N			n	Amides
Q				
Y			Y	Tyrosine
C			C	Cysteine

Table S5. A list of 16 DMS dataset proteins for which Protein Wordwise achieved 100% predicted functional residue coverage

Protein name	UniProt ID	Sequence length	Number of functional residues / Sequence length	Number of predicted residues / Sequence length
A4_HUMAN_ (Seuma_2022)	P05067	770	0.055	0.983
BLAT_ECOLX_ (Firnberg_2014)	P62593	286	0.832	1.000
CCDB_ECOLI_ (Adkar_2012)	P62554	101	0.505	0.931
DNJA1_HUMAN_ (Tsuboyama_2023)	P31689	65	1.000	1.000
DYR_ECOLI_ (Thompson_2019)	P0ABQ4	159	0.925	1.000
GAL4_YEAST_ (Kitzman_2015)	P04386	881	0.073	0.839
TADBP_HUMAN_ (Bolognesi_2019)	Q13148	414	0.200	0.995
MK01_HUMAN_ (Brenan_2016)	P28482	360	0.939	1.000
PTEN_HUMAN_ (Matreyek_2021)	P60484	403	0.868	1.000
PTEN_HUMAN_ (Mighell_2018)	P60484	403	0.988	1.000
RL40A_YEAST_ (Roscoe_2013)	P0CH08	128	0.531	1.000
RL40A_YEAST_ (Roscoe_2014)	P0CH08	128	0.555	1.000
SRC_HUMAN_ (Ahler_2019)	P12931	536	0.407	0.953
SRC_HUMAN_ (Chakraborty_2023)	P12931	536	0.405	0.924
SRC_HUMAN_ (Nguyen_2022)	P12931	536	0.388	0.924
UBC9_HUMAN_ (Weile_2017)	P63279	159	0.893	0.994

Table S6. An overview of datasets comprising the PWNet data resource for functional residue prediction

Dataset	Average label ratio	Sequence number	Mean coverage	Median coverage	Ref.
DNA-binding	0.083	964	0.795	0.921	CLAPE-DB
Small molecule-binding	0.033	4888	0.870	1.000	CLAPE-SMB
Metal ion-binding	0.015	3485	0.872	1.000	This work
ATP-binding	0.050	390	0.838	0.917	E2EATP
RNA-binding	0.186	915	0.774	0.833	CLAPE
Peptide-binding	0.030	3410	0.794	1.000	This work
PPI homo.	0.284	5649	0.709	0.727	DIPS-Plus
PPI hetero.	0.219	2591	0.734	0.778	DIPS-Plus
Enzyme active site	0.015	943	0.861	1.000	EasIFA
Ion channel	0.075	95	0.934	1.000	This work

Table S7. GO terms used in the ExpGO65 dataset

Function label	GO
ATP-dependent activity	GO: 0140657
Antioxidant activity	GO: 0016209
Binding	GO: 0005488
Purine ribonucleoside triphosphate binding	GO: 0035639
ATP binding	GO: 0005524
GTP binding	GO: 0005525
Amide binding	GO: 0033218
Antigen binding	GO: 0003823
Carbohydrate binding	GO: 0030246
Carbohydrate derivative binding	GO: 0097367
Chromatin binding	GO: 0003682
Hormone binding	GO: 0042562
Lipid binding	GO: 0008289
Nucleic acid binding	GO: 0003676
DNA binding	GO: 0003677
RNA binding	GO: 0003723
Organic cyclic compound binding	GO: 0097159
Peptide binding	GO: 0042277
Protein binding	GO: 0005515
Protein-containing complex binding	GO: 0044877
Small molecule binding	GO: 0036094
Sulfur compound binding	GO: 1901681
Catalytic activity	GO: 0003824
Cyclase activity	GO: 0009975
Demethylase activity	GO: 0032451
Hydrolase activity	GO: 0016787
Ribonucleoside triphosphate phosphatase activity	GO: 0017111
ATP hydrolysis activity	GO: 0016887

GTPase activity	GO: 0003924
Isomerase activity	GO: 0016853
Ligase activity	GO: 0016874
Lyase activity	GO: 0016829
Oxidoreductase activity	GO: 0016491
Transferase activity	GO: 0016740
Catalytic activity, acting on a nucleic acid	GO: 0140640
Catalytic activity, acting on a protein	GO: 0140096
Electron transfer activity	GO: 0009055
Molecular adaptor activity	GO: 0060090
Protein-macromolecule adaptor activity	GO: 0030674
Molecular carrier activity	GO: 0140104
Nucleocytoplasmic carrier activity	GO: 0140142
Molecular transducer activity	GO: 0060089
Cytoskeletal motor activity	GO: 0003774
Molecular sequestering activity	GO: 0140313
Protein folding chaperone	GO: 0044183
Receptor activity	GO: 0038024; GO: 0038023
Cargo receptor activity	GO: 0038024
Signaling receptor activity	GO: 0038023
Regulator activity	GO: 0045182; GO: 0140110<br>GO: 0098772; GO: 0140299
Molecular function regulator activity	GO: 0098772; GO: 0140299
ATPase regulator activity	GO: 0060590
Enzyme regulator activity	GO: 0030234
Small molecule sensor activity	GO: 0140299
Signaling receptor regulator activity	GO: 0030545
Transporter regulator activity	GO: 0141108
Transcription regulator activity	GO: 0140110
DNA-binding transcription factor activity	GO: 0003700
Transcription coregulator activity	GO: 0003712

Translation regulator activity	GO: 0045182
Structural molecule activity	GO: 0005198
Transporter activity	GO: 0005215
Lipid transporter activity	GO: 0005319
Transmembrane transporter activity	GO: 0022857
Carrier activity	GO: 0022857<br>(excluding GO: 0015267)
Channel activity	GO: 0015267

Table S8. Prediction performance (functional MCCs) for Word2Function for ExpGO65

Function label	Sequence ratio	Sequence number	Functional MCC
ATP-dependent activity	0.012	654	0.510
Antioxidant activity	0.006	307	0.538
Binding	0.753	42401	0.454
Purine ribonucleoside triphosphate binding	0.015	821	0.143
ATP binding	0.010	565	0.140
GTP binding	0.005	275	-0.001
Amide binding	0.006	347	0.280
Antigen binding	0.002	90	0.746
Carbohydrate binding	0.005	292	0.183
Carbohydrate derivative binding	0.027	1541	0.111
Chromatin binding	0.015	855	0.169
Hormone binding	0.003	172	0.191
Lipid binding	0.020	1107	0.306
Nucleic acid binding	0.141	7916	0.537
DNA binding	0.084	4715	0.582
RNA binding	0.059	3336	0.487
Organic cyclic compound binding	0.176	9903	0.451
Peptide binding	0.005	278	0.346
Protein binding	0.638	35911	0.425
Protein-containing complex binding	0.046	2580	0.132
Small molecule binding	0.082	4613	0.221
Sulfur compound binding	0.006	349	0.144
Catalytic activity	0.319	17973	0.722
Cyclase activity	0.001	48	0.378
Demethylase activity	0.001	67	0.617
Hydrolase activity	0.098	5538	0.708
Ribonucleoside triphosphate phosphatase activity	0.012	656	0.263
ATP hydrolysis activity	0.006	308	0.237

GTPase activity	0.006	340	0.198
Isomerase activity	0.012	654	0.690
Ligase activity	0.012	656	0.763
Lyase activity	0.019	1072	0.656
Oxidoreductase activity	0.058	3250	0.751
Transferase activity	0.127	7174	0.713
Catalytic activity, acting on a nucleic acid	0.027	1537	0.651
Catalytic activity, acting on a protein	0.088	4931	0.627
Electron transfer activity	0.004	203	0.384
Molecular adaptor activity	0.026	1467	0.114
Protein-macromolecule adaptor activity	0.023	1266	0.138
Molecular carrier activity	0.003	163	0.171
Nucleocytoplasmic carrier activity	0.001	31	0.707
Molecular transducer activity	0.028	1569	0.691
Cytoskeletal motor activity	0.001	63	0.566
Molecular sequestering activity	0.002	136	0.297
Protein folding chaperone	0.002	103	0.426
Receptor activity	0.028	1577	0.663
Cargo receptor activity	0.001	63	0.471
Signaling receptor activity	0.027	1523	0.674
Regulator activity	0.098	5493	0.372
Molecular function regulator activity	0.047	2646	0.288
ATPase regulator activity	0.002	95	0.603
Enzyme regulator activity	0.029	1637	0.253
Small molecule sensor activity	0.002	104	0.632
Signaling receptor regulator activity	0.010	579	0.421
Transporter regulator activity	0.003	189	0.093
Transcription regulator activity	0.048	2699	0.461
DNA-binding transcription factor activity	0.038	2158	0.498
Transcription coregulator activity	0.010	539	0.208
Translation regulator activity	0.004	221	0.515

Structural molecule activity	0.017	962	0.676
Transporter activity	0.061	3407	0.811
Lipid transporter activity	0.004	207	0.343
Transmembrane transporter activity	0.058	3258	0.805
Carrier activity	0.039	2174	0.812
Channel activity	0.019	1084	0.771

Sequence ratio refers to the proportion of sequences possessing a particular function relative to the total number of sequences. Sequence number indicates the absolute count of sequences exhibiting this function.

Table S9. Prediction performance (functional MCCs) for WordTableGO65 for ExpGO65

Function label	Sequence ratio	Sequence number	Functional MCC
ATP-dependent activity	0.012	654	0.619
Antioxidant activity	0.006	307	0.685
Binding	0.753	42401	0.457
Purine ribonucleoside triphosphate binding	0.015	821	0.307
ATP binding	0.010	565	0.267
GTP binding	0.005	275	0.344
Amide binding	0.006	347	0.371
Antigen binding	0.002	90	0.667
Carbohydrate binding	0.005	292	0.347
Carbohydrate derivative binding	0.027	1541	0.250
Chromatin binding	0.015	855	0.292
Hormone binding	0.003	172	0.391
Lipid binding	0.020	1107	0.384
Nucleic acid binding	0.141	7916	0.482
DNA binding	0.084	4715	0.537
RNA binding	0.059	3336	0.506
Organic cyclic compound binding	0.176	9903	0.421
Peptide binding	0.005	278	0.377
Protein binding	0.638	35911	0.455
Protein-containing complex binding	0.046	2580	0.259
Small molecule binding	0.082	4613	0.330
Sulfur compound binding	0.006	349	0.154
Catalytic activity	0.319	17973	0.665
Cyclase activity	0.001	48	0.534
Demethylase activity	0.001	67	0.771
Hydrolase activity	0.098	5538	0.727
Ribonucleoside triphosphate phosphatase activity	0.012	656	0.352
ATP hydrolysis activity	0.006	308	0.250

GTPase activity	0.006	340	0.432
Isomerase activity	0.012	654	0.723
Ligase activity	0.012	656	0.802
Lyase activity	0.019	1072	0.736
Oxidoreductase activity	0.058	3250	0.810
Transferase activity	0.127	7174	0.759
Catalytic activity, acting on a nucleic acid	0.027	1537	0.748
Catalytic activity, acting on a protein	0.088	4931	0.691
Electron transfer activity	0.004	203	0.403
Molecular adaptor activity	0.026	1467	0.196
Protein-macromolecule adaptor activity	0.023	1266	0.206
Molecular carrier activity	0.003	163	0.190
Nucleocytoplasmic carrier activity	0.001	31	0.353
Molecular transducer activity	0.028	1569	0.762
Cytoskeletal motor activity	0.001	63	0.640
Molecular sequestering activity	0.002	136	0.246
Protein folding chaperone	0.002	103	0.210
Receptor activity	0.028	1577	0.122
Cargo receptor activity	0.001	63	0.769
Signaling receptor activity	0.027	1523	0.666
Regulator activity	0.098	5493	0.765
Molecular function regulator activity	0.047	2646	0.422
ATPase regulator activity	0.002	95	0.397
Enzyme regulator activity	0.029	1637	0.539
Small molecule sensor activity	0.002	104	0.311
Signaling receptor regulator activity	0.010	579	0.707
Transporter regulator activity	0.003	189	0.581
Transcription regulator activity	0.048	2699	0.109
DNA-binding transcription factor activity	0.038	2158	0.505
Transcription coregulator activity	0.010	539	0.580
Translation regulator activity	0.004	221	0.237

Structural molecule activity	0.017	962	0.631
Transporter activity	0.061	3407	0.724
Lipid transporter activity	0.004	207	0.798
Transmembrane transporter activity	0.058	3258	0.515
Carrier activity	0.039	2174	0.799
Channel activity	0.019	1084	0.811

Sequence ratio refers to the proportion of sequences possessing a particular function relative to the total number of sequences. Sequence number indicates the absolute count of sequences exhibiting this function.

Table S10 | Comprehensive performance evaluation and ablation analysis on the ExpGO65 dataset.

	AUC	AP	Accuracy	Precision	Recall	F1 Score	MCC
**Raw ESM embedding**	0.705	0.288	0.969	0.526	**0.435**	0.463	0.427
**Word2Function**	0.688	0.295	0.969	0.587	0.400	0.450	0.424
**WordTableGO65**	**0.710**	**0.412**	**0.980**	**0.901**	0.431	**0.545**	**0.498**
**PROSITE**	0.550	0.104	0.947	0.534	0.111	0.135	0.147

Table S11 | Prediction performance (functional MCC) by number of functional annotation words.

Functional annotation word number	Functional MCC
0-2	0.160
3-5	0.313
6-8	0.577

Table S12 | Prediction performance (functional MCC) comparison across methods at different similarity thresholds

	50%	30%	10%
**GPSFun**	0.444	0.395	0.381
**GPSFun+WordTableGO65**	**0.457**	**0.467**	**0.459**
**PROSITE**	0.257	-0.088	-0.100
**Word2Function**	0.183	0.176	0.174
**WordTableGO65**	0.330	0.335	0.355
**ProtNote**	0.378	0.192	0.119
**ProtNote+WordTableGO65**	0.397	0.239	0.170
